# Correction: GGCX and VKORC1 inhibit osteocalcin endocrine functions

**DOI:** 10.1083/jcb.20140911104082019c

**Published:** 2019-04-30

**Authors:** Mathieu Ferron, Julie Lacombe, Amélie Germain, Franck Oury, Gérard Karsenty

Vol. 208, No. 6, March 16, 2015. 10.1083/jcb.201409111.

The authors noticed a mistake in the labeling of the genotyping primers in Fig. S1 A and Table S1. In Fig. S1 A, the primers P1 and P2 are actually flanking the 5′loxP site region. In addition, the P3 primer should appear downstream of the 3′loxP site. In Table S1, the names of the *Ggcx* genotyping primers P2 and P3 were swapped. Because of these mistakes, the genotyping PCR presented in Fig. S1 C, and in the bottom gels of Figs. 1 C and 4 A, were incorrectly labeled as 3′lox PCR instead of 5′lox PCR.

The authors apologize for this error, which does not affect in any way the conclusions of Figs. S1 C, 1 C, or 4 A, or any other data presented in the paper.

Both the HTML and PDF versions of the article have been corrected. These errors appears only in print and in PDF versions downloaded on or before April 29, 2019.

